# Correlation between Insulin Resistance and Microalbuminuria Creatinine Ratio in Postmenopausal Women

**DOI:** 10.1155/2022/9583611

**Published:** 2022-08-29

**Authors:** Han Na, Rong Wang, Hai-Long Zheng, Xiao-Pan Chen, Lin-Yang Zheng

**Affiliations:** ^1^Department of Endocrinology, The First Affiliated Hospital of Hainan Medical University, Haikou 571000, China; ^2^Department of Wound Repair, The First Affiliated Hospital of Hainan Medical University, Haikou 571000, China

## Abstract

**Objective:**

To study the relationship between insulin resistance and urinary microalbumin creatinine ratio in postmenopausal women.

**Methods:**

The selected research group comprised 104 postmenopausal women with type 2 diabetes who were admitted to the Department of Endocrinology in the green card center at the First Affiliated Hospital of Hainan Medical University between 2017 and 2019 inclusive. Ninety-eight postmenopausal women with the normal blood glucose metabolism hospitalized in the same period were used as the control group. The age, body mass index, systolic blood pressure (SBP), diastolic blood pressure (DBP), heart rate, fasting blood glucose, fasting insulin (FINS), glycosylated hemoglobin (HbA1c), total cholesterol (TC), triglyceride (TG), high-density lipoprotein (HDL-C), low-density lipoprotein (LDL-C), and urinary albumin-creatinine ratio (UACR) were analyzed. The insulin resistance index (HOMR-IR) was calculated, and the correlation between IR and UACR was analyzed.

**Results:**

Levels of HOMA-IR, SBP, HbA1c, HDL-C, LDL-C, TC, TG, FPG, FINS, and UACR in the study group were higher than those in the control group, and a significant difference was found between the groups (*P* < 0.05). The level of DBP in the study group was lower than that in the control group, and the difference was statistically significant (*P* < 0.05). Pearson correlation analysis showed that UACR was positively correlated with HOMA-IR and HbA1c (*r* = 0.254, *r* = 0.565, *P* < 0.01). Multiple linear stepwise regression analysis further showed that HOMA-IR and age were positively correlated with UACR (*P* < 0.05).

**Conclusion:**

There is a correlation between IR and UACR in postmenopausal women. IR is an independent risk factor for UACR.

## 1. Introduction

After menopause, as estrogen levels in women decrease, the prevalence of metabolic syndrome (MetS) mainly including insulin resistance (IR), type 2 diabetes, hyperlipidemia, and hypertension gradually increases, ranging from 16.9% to 69.0% across different populations [1, 2]. There is evidence to suggest that MetS significantly increases the prevalence and severity of menopausal symptoms [3]. It has also been shown that serum lipid levels, such as total cholesterol (TC), low-density lipoprotein-cholesterol (LDL-C), triglyceride (TG), and apolipoprotein B (Apo B), are increased during the one-year interval before and after the last menstrual period and that these parameters are associated with changes caused by menopause [4, 5]. Lower estrogen levels are thought to be one of the causes of ovarian hormone changes, irregular menstrual cycles, and the development of cardiovascular disease (CVD); however, estrogen also has a positive effect on the regulation of insulin sensitivity [6–8]. IR refers to impaired insulin action in glucose uptake, utilization, and other aspects, that is, the biological effect of a certain amount of insulin is lower than the expected normal level, and the body develops compensatory hyperinsulinemia to overcome IR [9]. Studies have shown that postmenopausal women are at high risk of developing IR [10], and IR has moreover become a major factor in increased mortality among patients with diabetes and cardiovascular disease. The presence of microalbuminuria (MAU) reflects the state of endothelial damage in the whole body; MAU can predict the development of microvascular events, such as diabetic nephropathy (DN), as well as macrovascular events. In this study, the HOMA-IR index was used to assess IR, and the urine albumin-creatinine ratio (UACR) was used as an indicator of MAU. The correlation between IR and UACR was analyzed by investigating postmenopausal women who visited our hospital. This enabled us to understand the correlation between IR and UACR levels in a specific population of postmenopausal female patients, as well as to provide new clinical evidence for the study of IR and UACR.

## 2. Materials and Methods

### 2.1. Study Subjects

The selected research group comprised 104 postmenopausal women with type 2 diabetes who were admitted to the Department of Endocrinology in the green card center at the First Affiliated Hospital of Hainan Medical University between 2017 and 2019. Ninety-eight postmenopausal women with normal glucose metabolism hospitalized in the same period were selected as the control group. The total number of cases in this study was 202.

Inclusion criteria: menopause or menopause for more than 12 months at the time of admission, aged over 40 years old, previous regular menstruation (average cycle of 25–35 days), and diagnosis of T2DM in accordance with the 2020 Guidelines for Diagnosis and Staging of Diabetes Mellitus, revised by the Chinese Medical Association, and the control group with normal oral glucose tolerance test [11].

Exclusion criteria: patients who had undergone hysterectomy or bilateral oophorectomy, patients who had received hormone replacement therapy before admission, patients with a history of polycystic ovary syndrome, patients who had received insulin for blood glucose control and/or application of ACEI and ARB drugs before admission, malignant tumors, and patients with breast cancer, thrombotic diseases, and liver and kidney insufficiency. This study was approved by the hospital ethics committee, and the included subjects were informed and signed a consent form.

According to the insulin resistance index HOMA-IR, the study group was divided into three subgroups: IR-L group (*n* = 34), IR-M group (*n* = 35), and IR-H group (*n* = 33).

### 2.2. Definition of Indicators

#### 2.2.1. HOMA-IR Calculation

Fasting blood glucose (FPG) and fasting insulin (FINS) were measured in both groups. The IR index was calculated using the insulin homeostasis model with the formula HOMA-IR = FPG (mmol/l) × FINS (mU/L)/22.5.

### 2.3. UACR Measurement

The first morning urine or the second morning urine of subjects was taken, after which MAU was determined via rate nephelometry using a Roche DDP automatic biochemical analyzer; urine creatinine was assessed using an automatic biochemical detector (sarcosine oxidase method); UACR (mg/g) = MAU/urine creatinine.

### 2.4. Observation Indicators

Age, body mass index (BMI), systolic blood pressure (SBP), diastolic blood pressure (DBP), FPG, 2hPG, FINS, HbA1c, TC, TG, HDL-C, LDL-C, UACR, and HOMA-IR were observed. Natural logarithms of HOMA-IR were taken and then statistically analyzed.

### 2.5. Statistical Methods

All data were analyzed and processed using the SPSS 22.0 statistical package. The normal distribution of measurement data was expressed as $\bar{x}$ ± *s*, while the nonnormal data were expressed as M (Q25–Q75). An independent samples *t*-test was used to compare the means of the groups, and a one-way analysis of variance (one-way ANOVA) was used for comparison between multiple groups. Pearson correlation analysis was employed to analyze the association between HOMA-IR and UACR, and multiple linear regression was used to analyze the related influencing factors of UACR. Nonnormally distributed data were log-transformed to facilitate statistical analysis. Rates were compared using the *χ*^**2**^ test. *P* < 0.05 was considered a statistically significant difference.

## 3. Results

### 3.1. General Clinical Characteristics of the Study Subjects

There was no statistically significant difference in age, BMI, HDH-C, LDL-C, TC, and TG levels between the study group and control group (*P* > 0.05), indicating that the baseline was aligned and the groups were comparable. The levels of HOMA-IR, SBP, HbA1c, HDL-C, LDL-C, TC, TG, FPG, FINS, and UACR in the study group were higher than those in the control group, and the differences between the groups were statistically significant (*P* < 0.05). The levels of DBP in the study group were lower than those in the control group, and the differences were statistically significant (*P* < 0.05). Further details are given in Table 1.

### 3.2. Comparison of General Information and Laboratory Test Indexes in the Three Study Groups

#### 3.2.1. Comparison of General Clinical Information in the Three Study Groups

The differences in age, HbA1c, and TC among the three groups were statistically significant (*P* < 0.05), indicating that these differences were statistically significant in at least two groups. A two-by-two comparison was performed, and the differences were found to be statistically significant (*P* < 0.01) in the IR-L group compared with the IR-H group, as well as in the IR-M group compared with the IR-H group (*P* < 0.05). Further details are given in Table 2.

#### 3.2.2. Comparison of UACR in the Three Study Groups

As HOMA-IR increased, UACR also increased. The difference when comparing the UACR levels of the three groups was statistically significant (*P*=0.000). Furthermore, when the three groups were compared in a two-by-two manner, the difference between the IR-L group and the IR-H group was statistically significant (*P*=0.001), as was that between the IR-M group and the IR-H group (*P*=0.000). Further details are given in Table 3.

### 3.3. Correlation Analysis of HOMA-IR and UACR

Pearson correlation analysis showed that UACR was positively correlated with HOMA-IR and HbA1c (*r* = 0.254, *r* = 0.565, *P* < 0.01), as given in Table 4.

### 3.4. Multiple Linear Regression

Multiple linear stepwise regression analysis was performed with UACR as the dependent variable, while age, BMI, systolic blood pressure, diastolic blood pressure, HbA1c, HDL-C, LDL-C, TC, TG, and HOMA-IR were the independent variables. The results showed that HOMA-IR and age entered the equation, and the regression equation was obtained as UACR = 0.975 + 0.020 age +3.184 HOMA-IR. Age and UACR were positively correlated (*P* < 0.05), as given in Table 5.

## 4. Discussion

Studies have shown that the presence of MAU in the overall population reflects the status of systemic vascular endothelial damage [12, 13]. Moreover, the MAU level is one of the most important indicators of the severity and prognosis of type 2 diabetic patients and has accordingly been clinically applied to diagnose and predict DN [13]. Menopause is a physiological change unique to women. One-third of a woman's life will be spent in the menopausal state, during which the sex hormones secreted by the ovaries are significantly reduced due to the decline of ovarian function [9]. Numerous studies have found that following the onset of menopause, the incidence of diabetes and other complications occur at a significantly higher rate in women than in men, suggesting that postmenopausal women have IR and insulin secretion disorders [14]; moreover, the proportion of DN-induced end-stage renal disease is significantly higher in postmenopausal women [15]. IR is a state in which peripheral target organs are both less sensitive and less responsive to endogenous or exogenous insulin, meaning that the body secretes too much insulin to maintain blood glucose stability, resulting in hyperinsulinemia. The occurrence of IR involves multiple signaling pathways; it is also a state in which multiple sites and levels of insulin signaling act together abnormally [16], which is one [12, 17] of the independent risk factors for kidney injury and renal failure. Studies have confirmed that IR is already present in the early stages of DN and gradually worsens as DN progresses. IR has become a major factor contributing to the increased relative risk of cardiovascular disease and cardiovascular disease mortality in DN patients [18].

In this study, it was found that HbA1c, FPG, FINS, and UACR levels were higher in the study group than in the control group, indicating abnormal glucose metabolism parameters among postmenopausal women with diabetes. The UACR levels in the study group were much higher than those in the control group, indicating that postmenopausal women with combined diabetes mellitus are mainly characterized by elevated UACR levels, a finding that can provide a strong basis for clinical treatment and prevention. The data from the study group of postmenopausal women in the present research showed significantly increased levels of UACR with increasing HOMA-IR in the three HOMA-IR groups, with statistically significant differences between groups: Pearson correlation analysis showed that HOMA-IR and UACR were positively correlated; multiple linear stepwise regression analysis further showed that HOMA-IR and UACR were positively correlated, suggesting that HOMA-IR is a risk factor for UACR in postmenopausal T2DM patients. It was also confirmed that podocytes in the glomerular filtration barrier are insulin-sensitive cells, and insulin-specific antibodies have appeared on podocyte since the early stage of diabetes. Podocyte injury is positively correlated with IR, and MAU gradually appears as podocyte injury increases, and podocytes play a key role in the progression of DN [19, 20]. IR and its associated hyperinsulinemia can upregulate renin-angiotensin system (RAS) activity, which increases angiotensin II (AngII) in circulation; this increased AngII can exacerbate IR in diabetic patients through multiple pathways. Hyperinsulinemia stimulates increased expression of the type 1 receptor (AT1R) for tethered angiotensin II (AngII), which is found in afferent arterioles, efferent arterioles, glomerular cells, and tubules. This makes the kidney more susceptible to activated RAAS compared to other organs, putting the glomerulus in a hyperfiltration state, damaging glomerular vascular endothelial cells, and leading to early renal lesions and early microalbuminuria [21].

This study was inconsistent with the study results of De Cosmo et al. [22], who included 363 white male and 349 white female patients diagnosed with type 2 diabetes after the age of 30. These patients were divided into groups without MAU microalbuminuria or macroalbuminuria in male and female patients, respectively, and had a higher risk of urinary albumin in the third quartile of IR than in the first quartile of IR in male patients (Q3: OR 2.2 (95% CI 1.2–4.2)); (Q4: OR 4.1 (2.2–7.9)). It was also found that HOMA-IR was significantly associated with urinary albumin in male patients with type 2 diabetes, but not in female patients. The reason for this discrepancy with our findings may relate to differences in the subjects of the observational studies: the population of this study was postmenopausal diabetic women, while the study population of De Cosmo et al. [23] was individuals with type 2 diabetes. Studies have shown that estrogen levels are significantly lower in postmenopausal women due to ovarian atrophy. It has also been shown that IR levels in postmenopausal women are 44%, demonstrating that estrogen reduction plays a role in the development of IR in postmenopausal women [12]. There are several potential mechanisms by which estrogen reduction could cause IR. (1) The increase of cytoplasmic free calcium level may be the basis of IR. The cytoplasmic calcium concentration affects the postinsulin receptor effect and impairs the glucose utilization of insulin target cells. Estrogen, as a calcium channel blocker, can reduce the cytoplasmic free calcium level. The decrease of estrogen level after menopause can thus lead to the increase of cytoplasmic calcium, thereby reducing the insulin effect and glucose utilization, resulting in IR. (2) Sex hormone binding globulin (SHBG) is associated with many T2DM risk factors. Plasma estrogen induces SHBG production in the liver, which is significantly lower in postmenopausal women compared with premenopausal women; this decrease in SHBG levels is not only associated with IR but is also an independent risk factor for the development of diabetes. Studies have shown that elevated SHBG levels are effective in preventing the development of IR [24]. (3) Lower estrogen levels after menopause also cause redistribution of body fat, and the physiological effects of insulin are reduced when abundant adipocytes are hyporesponsive to insulin and IR occurs [25].

In conclusion, in postmenopausal women with type 2 diabetes, the levels of UACR increased with the aggravation of IR, while estrogen levels decreased. A correlation was observed between IR and UACR; moreover, IR is an independent risk factor for UACR, providing a strong basis for clinical diagnosis and treatment. However, this study is cross-sectional and lacks follow-up, meaning that the causal relationship between IR and UACR levels in postmenopausal women cannot be clarified at present.

## Figures and Tables

**Table 1:** Comparison of general information between two groups of patients ($\bar{x}$*± s*).

Indicators	Study group (*n* = 104)	Control group (*n* = 98)	*t*	*P*
Age	58.90 ± 7.77	57.12 ± 3.04	2.169	0.032
HOMA-IR	5.32 ± 6.29	1.99 ± 1.03	−5.16	<0.0001^*∗*^
BMI	23.99 ± 4.25	24.82 ± 3.33	−1.531 to −2.33	0.126
SBP	134.85 ± 20.47	128.77 ± 16.82	2.94	0.023^*∗*^
DBP	73.62 ± 12.23	78.38 ± 10.66	13.884	0.004^*∗*^
HbA1c	9.04 ± 2.34	5.72 ± 0.29	1.063	<0.0001^*∗*^
HDL-C	1.30 ± 0.31	1.34 ± 0.27	−1.494	0.289
LDL-C	3.38 ± 1.57	3.11 ± 0.76	−1.292	0.130
TC	5.62 ± 1.37	5.40 ± 0.96	−2.134	0.198
TG	1.77 ± 1.26	1.46 ± 0.72	10.164	0.340
FPG	10.27 ± 4.64	5.47 ± 0.50	3.009	<0.0001^*∗*^
FINS	11.38 ± 10.22	8.06 ± 3.88	−5.577	0.003^*∗*^
UACR	236.54 ± 406.55	7.45 ± 5.29		<0.0001^*∗*^

^*∗*^ *P* < 0.05.

**Table 2:** Comparison of general clinical information in three HOMA-IR groups.

	HOMA-IR				
	IR-L (*n* = 34)	IR-M (*n* = 35)	IR-H (*n* = 33)	F	*P*
Age	62.41 ± 10.78	62.97 ± 9.93	59.40 ± 8.17	3.377	0.038^*∗*^
BMI	24.26 ± 5.63	24.05 ± 3.30	23.698 ± 3.6	0.159	0.853
SBP	130.59 ± 20.40	140.97 ± 23.77	132.89 ± 15.49	2.536	0.084
DBP	72.41 ± 16.04	75.03 ± 11.35	73.40 ± 8.52	0.399	0.672
HbA1c	7.85 ± 2.16	8.77 ± 1.83	10.46 ± 2.30	13.634	<0.0001^*∗*^
HDL-C	1.24 ± 0.33	1.32 ± 0.31	1.33 ± 0.30	0.939	0.394
LDL-C	3.13 ± 0.85	3.29 ± 0.84	3.36 ± 0.89	1.834	0.165
TC	5.13 ± 1.16	5.87 ± 1.24	5.85 ± 1.58	3.402	0.037^*∗*^
TG	1.76 ± 1.04	1.61 ± 0.95	1.96 ± 1.68	0.710	0.494

^*∗*^ *P* < 0.05.

**Table 3:** Comparison of UACR in three HOMA-IR groups.

	IR-L (*n* = 34)	IR-M (*n* = 35)	IR-H (*n* = 33)	*F*	*P*
UACR	2.908 ± 1.21	4.357 ± 0.677^*∗*^	5.043 ± 1.004^*∗*^^#^	40.676	<0.0001

Compared with IR-L, ^*∗*^*P* < 0.05; compared with IR-M, ^#^*P* < 0.05.

**Table 4:** Correlation analysis between UACR and individual indicators; UACR was compared after taking the natural logarithm.

Influencing factors	*R*	*P*
HbAlc	0.254	0.009
HOMA-IR	0.565	0.000

**Table 5:** Multiple linear stepwise regression with UACR as the dependent variable.

Items	*β*	*T*	*P*
Constants	0.975	1.393	0.167
Age	0.20	2.010	0.047
HOMA-IR	3.184	9.669	0.000

## Data Availability

The data generated or analyzed during this study are included within this article.
